# Nanostructured Materials Based on Noble Metals for Advanced Biological Applications

**DOI:** 10.3390/nano9111593

**Published:** 2019-11-10

**Authors:** Iole Venditti

**Affiliations:** Sciences Department, Roma Tre University, via della Vasca Navale 79, 00146 Rome, Italy; iole.venditti@uniroma3.it; Tel.: +39-06-5733-3388

**Keywords:** nanomaterials, noble metal nanoparticles, gold nanomaterials, silver nanomaterials, hybrid metal–polymer nanoparticles, nanomedicine, biotechnological applications, nanomaterials for drug delivery, nanomaterials for sensing

## Abstract

This special issue focuses on highlighting the progress of last decade regarding the new nanostructured materials based on noble metals, especially gold and silver. Innovative preparations, functionalizations, and characterizations of these nanomaterials are investigated. Moreover, biotechnological applications, and advanced uses of these compounds for environmental sensing are reported. In particular gold and silver nanomaterials are widely studied due to their high stability, amazing chemical–physical features and, for silver, marked antibacterial properties. It is also hoped that the current special issue will encourage multidisciplinary research on noble metal nanomaterials, expanding the range of potential biological applications. This must be associated with improvements in synthetic methods and with economic feasibility studies of the proposed processes, also exploring the ecotoxicological aspects.

Nanostructured materials based on noble metal nanoparticles (NPs) allow synergistic enhancement of their functional properties due to the low dimensionality. In fact, nanodimension is the strategic key for a wide range of bio-applications, such as biosensors, biocatalysis, drug delivery, imaging, and theranostic applications [1,2,3,4,5]. A huge variety of new materials and composites have been improved, mainly via chemical approaches, using metal surface engineering to build new synergic hybrid systems, in particular based on gold and silver nanoparticles (AuNPs, AgNPs) [6,7,8].

This special issue focuses on highlighting the progress of new nanostructured materials, based on noble metals, their preparation, functionalization, characterization, and advanced application in biological fields. In fact, in this last decade, gold and silver nanoparticles have been widely used in advanced biological technologies, thanks to the high stability of the former and to the marked antibacterial properties of the latter.

Burdușel et al. [9], make a review about nanostructured silver compounds intensively explored for unconventional and enhanced biomedical applications, thanks to their size-related attractive physicochemical properties and biological functionality, including their high antimicrobial efficiency and non-toxic nature. AgNP-based nanosystems and nanomaterials are suitable alternatives for drug delivery, wound dressing, tissue scaffold, and protective coating applications. Various physicochemical parameters were related to the intrinsic antimicrobial effects exhibited by AgNPs, such as size, shape, concentration, surface charge, and colloidal state. Moreover, the impressive available surface of nanosilver allows the coordination of many ligands, thus enabling tremendous possibilities with respect to the surface functionalization of AgNPs.

There is a significant amount of research data proving the beneficial effects of AgNPs in novel biocompatible and nanostructured materials and devices developed for modern therapeutic strategies. In addition to their attractive and versatile antimicrobial potential, AgNPs provide additional mechanical, optical, chemical, and biological peculiarities that recommend them for the design, obtaining, evaluation, and clinical assessment of performance-enhanced biomaterials and medical devices. Still, thorough investigations regarding their short-term and long-term toxicity, as well as the responsible toxic-related mechanisms, are required.

The current limitations related to conventional healthcare practice and the latest challenges resulting from nanosilver-based technology outline the impressive potential of silver nanoparticles in biomedicinal applications. Whether we consider the modification of available biomaterials and devices or the development of novel nanostructured ones, AgNPs are ideal candidates for achieving the very close modern biomedicine desideratum.

Auría-Soro et al. present a review on the significant impact of nanotechnology on medicine [10]. The studies about the biological response to NPs are greatly investigated in parallel with nano-bio interactions, which have influenced NP design. Both were in concordance with the evolution of NPs for biomedical applications. Many studies have investigated and demonstrated that NPs can enter into the human system. Therefore, the NP characteristics on biological systems, such as their physicochemical properties (size, shape, surface, coating and morphology, surface charge, hydrophobicity, chemical composition, structure, and the state of agglomeration), the types of biomolecules present, and the bio-identity of NP protein corona are important issues to characterize in order to know how they interact with cells, organisms, biological medium, biomolecules, and other biological systems or even with other nanomaterials. These studies helped determine their possible biocompatibility and toxicity in biological micro-environments and to engineer nontoxic nanomaterials, which may be used in biomedical applications.

With the potentially wide application of NPs in the future, these may be extensively used in various fields, especially in immunotherapy for clinical diagnosis and therapy based on their size, biocompatibility, surface chemistry, and adjustable toxicity. Immunotherapy combined with nanomedicines may be used to treat different types of cancer due to their excellent efficacy in penetration, specific retention, and killing of tumor cells.

The human proteome study [11] can be an arduous and discouraging task due to the high number of proteins, encoded by around 25,000 different genes, from which multiple protein variants are generated by post-translational modifications. The concept of proteomics involves a comprehensive study on the structures, localizations, post-translational modifications, functions, and interactions of all proteins expressed by an organism at a certain time and under certain conditions. The nanotechnology field has been expanded by providing innovative methods capable of responding to proteomic demands. In this sense, nanotechnology applications in proteomics have established a novel technical platform termed “nanoproteomics.” Detection techniques without labels are useful in the study of protein interaction kinetics, thanks to avoiding steric impediments caused by the presence of labels. The design and development of new multi-functional platforms based on nanomedicine could be of great interest in the unlabeled detection of protein–protein interactions given the possibility of synthesizing de novo proteins “in vitro” in the presence of these nanosystems.

Jang et al. investigate a facile and effective shape-controlled synthesis of gold nanostructures and their photothermal therapeutic effect [12]. The described procedure involves the simple mixing of tetrachloroauric acid (HAuCl_4_) and Ethylenediaminetetraacetic acid tetrasodium salt (EDTA tetrasodium salt) in an aqueous solution at room temperature, without additional ligands or toxic reagents. Adjusting the molar ratios of HAuCl_4_ to EDTA tetrasodium salt enables effective morphology control of Au nanostructures from spheres to branched forms and nanowire networks. Detailed control experiments revealed that the four deprotonated carboxylic acids of the EDTA tetrasodium salt provided effective growth control and stabilization. The Au nanowire networks showed strong absorption in the near-infrared (NIR) region and hence were suitable for photothermal therapy. Under NIR irradiation, the Au nanowire networks allowed for selective destruction of cancerous human primary glioblastoma cells (U87MG cells) by local heating, generated by the NIR absorption. This work demonstrates the development of a simple synthetic route to NIR-active Au nanostructures, which can be extended to other applications including optical sensing and surface-enhanced Raman scattering.

For effective placement of target analytes on sensor surfaces and their monitoring, Lee et al. present a straightforward measurement method based on nanoplasmonic sensors of double-bent Au strip (DAS) arrays, multiple dip-coating of analytes, self-alignment of analytes in the region of strong plasmonic fields, and spectrometric monitoring of localized surface plasmon resonance (LSPR) peaks [13]. Using this method, closely packed polystyrene (PS) beads in the valleys of the DAS array and pH-dependent stability of the exosomes were successfully monitored in terms of shifts in the LSPR peaks. As a small amount of target analytes can accumulate in the plasmonic hot spot due to multiple dip-coating cycles, and the LSPR peak can be measured with a conventional UV-vis (ultraviolet–visible) spectrometer under physiological conditions, it is expected that the proposed measurement platform will be useful for studying the stability of various drug delivery vesicles and their efficiencies.

Fratoddi et al. prepare conjugates between strongly hydrophilic gold nanoparticles, AuNPs, and copper(I) complexes [14]. In particular, loading and release studies were performed using two different copper(I) antitumor complexes, namely [Cu(PTA)_4_]^+^[BF_4_]^−^ (A; PTA = 1,3,5-triaza-7-phosphadamantane) and [HB(pz)_3_Cu(PCN)] (B; HB(pz)_3_ = tris(pyrazolyl)borate, PCN = tris(cyanoethyl)phosphane). In the water-soluble compound A, the metal is tetrahedrally arranged in a cationic moiety, while compound B is a mixed-ligand (scorpionate/phosphane), neutral complex insoluble in water. Loading protocols and efficiency are also related to these structural aspects and were optimized to obtain loading efficiency η = 90 ± 4% and η = 65 ± 10%, respectively, for AuNPs-A and AuNPs-B. Structural differences of A and B induced different behaviors regarding the interactions with the gold surface, as showed by the high-resolution X-ray photoelectron spectroscopy (HR-XPS) studies. In fact, for compound A, nitrogen partially transfers electrons to the surface of the metal nanoparticles, creating an interaction that causes a slow release in water, less than 10% in 4 days. On the other hand, in B compound the N≡C−R groups hook onto the surface of the gold, producing a strong interaction that makes the release not appreciable in the same time interval (up to 4 days). Therefore, both AuNPs-A and AuNPs-B represent promising examples of water-soluble gold nanocarriers suitable to improve the bioavailability of synthetic drugs, especially considering the enhanced permeability and retention (EPR) effect of AuNPs. AuNPs-A, which achieved a slow release, opens the way for biological in vitro studies to explore the synergic activity of copper complexes and gold nanoparticles.

Rinaldi et al. load hydrophilic AgNPs in two different niosomes, producing two systems, namely NioTw20 + AgNPs and NioSp20 + AgNPs [15]. A deep physical–chemical characterization was carried out to obtain information on the influence of AgNPs on the preparation and features of niosomal formulations. First of all, the dynamic light scattering (DLS) studies confirm the nanosize and stability of both systems in water. Moreover, the entrapment efficiency for the two systems was investigated, and it was more efficient for Span 20 than Tw20 niosomes, which was probably related to their different internal structures. Microviscosity and polarity investigations demonstrated that no interactions occurred between the niosomal double layer and the AgNPs, which were probably located inside aqueous compartments. The small-angle X-ray scattering (SAXS) data confirmed the presence of the AgNPs located inside the aqueous compartment of the two niosomal systems, and also allowed highlighting the different structures of their double layers. The morphological characterization indicates that the niosomes maintained spherical shapes. Moreover, stability was confirmed in water, bovine serum, and human serum. Moreover, hydrophilic and lipophilic probe release profiles were obtained in 4-(2-hydroxyethyl)-1-piperazineethanesulfonic acid (HEPES) and in human serum. In conclusion, both systems evidenced the entrapment of AgNPs: NioTw20-AgNPs and NioSp20-AgNPs. The two systems are stable in water, bovine serum, and human serum, and maintain the ability to entrap also hydrophilic or lipophilic model molecules. This work demonstrates that the niosomes’ features are not altered by AgNPs loading and confirms that these niosomal formulations are good candidates for the delivery of AgNPs together with other drugs, opening new promising ways for their biotechnological applications.

In the last article of the special issue, silver nanoparticles are investigated as sensing materials for Hg^2+^ in water [16]. AgNPs were synthesized using hydrophilic capping agents, i.e., citrate (Cit) and L-cysteine (L-cys). The characterization by means of UV-vis, Fourier transform infrared spectroscopy (FTIR), HR-XPS and near edge X-ray absorption fine structure (NEXAFS) spectroscopies, confirmed the surface functionalization. Their nanodimensions were studied by DLS and transmission electron microscopy (TEM) analysis, showing diameter less than 10 nm. AgNPs showed high selectivity and sensitivity for Hg^2+^ in water (concentration range: 1–10 ppm) respect to 16 different metal ions investigated. The AgNPs-Hg^2+^ system was deeply investigated by means of DLS, inductively coupled plasma mass spectrometry (ICP-MS), TEM and HR-XPS. Measurements of Ag concentration in fresh and marine aqueous media showed low Ag^+^ ions release, probably due to the good Cit/L-cys covering, also confirmed by HR-XPS data. Moreover, AgNPs ecosafety was confirmed by ecotoxicity tests which showed no effects on algal growth of both freshwater *R. subcapitata* and marine *P. tricornutum* algae in the range of tested concentrations (10–500 mg/L). Our results further support the hypothesis that this specific coating of AgNPs prevent dissolution of Ag^+^ ions in both fresh and saltwater. These results open new ways for AgNPs sensing applications in environmental tests on more complex biological systems, up to tests on real environmental aquatic scenarios.

In conclusion, as editor of this special issue, I am aware that the diversity and innovation of new compounds and tools that are rapidly developing in the field of multidisciplinary research related to nanomaterials based on noble metals, cannot all be collected in a single volume. However, I am sure that this collection will contribute to the interest of research in this area, providing our readers with a broad and updated scenario on this topic.
